# Structure Analysis of *Entamoeba histolytica* DNMT2 (EhMeth)

**DOI:** 10.1371/journal.pone.0038728

**Published:** 2012-06-21

**Authors:** Eike C. Schulz, Heide M. Roth, Serge Ankri, Ralf Ficner

**Affiliations:** 1 Abteilung für Molekulare Strukturbiologie, Institut für Mikrobiologie und Genetik, Georg-August-Universität Göttingen, Göttingen, Germany; 2 Department of Molecular Microbiology, The Bruce Rappaport Faculty of Medicine, Technion, Haifa, Israel; Institut Pasteur, France

## Abstract

In eukaryotes, DNA methylation is an important epigenetic modification that is generally involved in gene regulation. Methyltransferases (MTases) of the DNMT2 family have been shown to have a dual substrate specificity acting on DNA as well as on three specific tRNAs (tRNA^Asp^, tRNA^Val^, tRNA^Gly^). *Entamoeba histolytica* is a major human pathogen, and expresses a single DNA MTase (EhMeth) that belongs to the DNMT2 family and shows high homology to the human enzyme as well as to the bacterial DNA MTase M.*Hha*I. The molecular basis for the recognition of the substrate tRNAs and discrimination of non-cognate tRNAs is unknown. Here we present the crystal structure of the cytosine-5-methyltransferase EhMeth at a resolution of 2.15 Å, in complex with its reaction product S-adenosyl-L-homocysteine, revealing all parts of a DNMT2 MTase, including the active site loop. Mobility shift assays show that *in vitro* the full length tRNA is required for stable complex formation with EhMeth.

## Introduction

In eukaryotes, DNA methylation is an important epigenetic mark that is only found at carbon 5 (C_5_) of cytosine and is generally involved in gene regulation. Upon methylation of CpG sites in promotor regions the gene expression is usually reduced. Furthermore, 5-cytosine methylation is considered to play a role in silencing of transposal and repetitive elements, in developmental processes, in X-chromosome inactivation, in genetic imprinting and to reduce transcriptional noise. Additionally, DNA methylation is also associated with diseases such as ICF- and Rett-syndrome as well as in the initiation and progression of various cancers [1], [2], [3], [4]. Methylation is carried out by 5-cytosine DNA-methyltransferases (5 mC DNA MTases; EC 2.1.1.37) that typically depend on S-adenosyl-L-methionine (AdoMet) as a cofactor [3], [5], [6].

Mechanistically, one of the best-studied 5 mC DNA MTases is *Haemophilus haemolyticus* M.*Hha*I, which also serves as a structural paradigm for other 5 mC DNA MTases [7], [8]. Briefly, for methylation, a cytosine is flipped out of the regular B-DNA in order to adopt an extra-helical position. Following a nucleophilic attack from a conserved cysteine, the cytosine is covalently attached to the enzyme. Subsequently, a highly conserved glutamate protonates N_3_ in the cytosine ring. This activates C_5_ and allows the abstraction of the methyl group of AdoMet. The methylation of C_5_ finally resolves the covalent complex and releases the methylated DNA and S-adenosyl-L-homocysteine (AdoHcy) from the active site [3], [7], [8], [9], [10], [11]. In the traditional model at least two different families of MTases were proposed to exist in eukaryotes: one family that is required for *de novo* methylation of DNA targeting un-methylated DNA, while the other family is specific for hemi-methylated DNA copying the methylation pattern in every replication cycle [2], [12]. In fact this traditional model holds mostly true, however, the *de novo* DNA nucleotide methyltransferases (DNMT3a/3b) and the maintenance DNA nucleotide methyltransferases (DNMT1) have partially overlapping functions and are supported by a number of other proteins [4], [13], [14]. The mechanism leading to *de novo* methylation of CpG sites was suggested based on the crystal structure of DNMT3a in complex with its inactive isoform DNMT3L [13], [15], [16]. Furthermore, two distinct crystal structures of DNMT1 in complex with DNA have been solved recently providing profound insights into substrate discrimination of hemi-methylated substrate DNA over un-methylated DNA, which is not targeted by DNMT1 [17], [18].

However, there is also a third family of eukaryotic DNA nucleotide methyltransferases (DNMT2) that is widely distributed and highly conserved [19], [20]. The role of DNMT2 was enigmatic for a long time, as initial studies did not recognize any enzymatic activity and knockout mutants showed only minor phenotypes [21], [22], [23]. Moreover, structural analysis of human DNMT2 showed a high similarity to M.*Hha*I and did not reveal structural reasons for the lack of MTase activity in DNMT2 [19]. Later, a weak but detectable DNA MTase activity could be assigned to DNMT2 from *Homo sapiens*, *Drosophila melanogaster* and *Dictyostelium discoideum* [24], [25], [26], [27]. Surprisingly, DNMT2 MTases are not only involved in the methylation of DNA but also in the methylation of tRNA^Asp^, tRNA^Val^ and tRNA^Gly^ at cytosine 38 of the tRNA [23], [28], [29]. Importantly, for tRNA methylation DNMT2 follows the reaction mechanism established for 5 mC DNA MTases. While RNA MTases utilize a cysteine residue from motif VI for the initial attack, it has been shown that, like 5 mC DNA MTases, DNMT2-enzymes use a cysteine residue from motif IV (Cys79 in DNMT2) as the catalytic nucleophile for the initial attack [3], [23], [30], [31]

The gastrointestinal, protozoan parasite *Entamoeba histolytica* is the causative agent of amoebiasis and annually leads to 50 million infections in humans of which 100.000 develop fatally [32], [33]. Documented DNA methylation in *E. histolytica* raised the question for a specific DNA MTase in the parasite. A BLAST search for DNA MTases in *E. histolytica* led to the identification of a 37.4 kDa DNA MTase (EhMeth) with high sequence homology to human DNMT2 [34]. Furthermore, it could be shown that inhibition of EhMeth by the general MTase inhibitor 5-azacytidine (5-AzaC) leads to a decreased virulence of *E. histolytica* trophozoites [34]. Since amoebiasis poses a major thread and 5-AzaC has a number of undesirable side effects, a species-specific EhMeth inhibitor would be an attractive asset in the treatment of amoebiasis [35].

In contrast to its human homolog, EhMeth was demonstrated to be responsible for the methylation of the genomic DNA of *E. histolytica* [34], [36]. The DNA elements that have been shown to be methylated in *E. histolytica* include ribosomal DNA, scaffold/matrix attachment regions (S/MAR), long interspersed nuclear elements (LINE) as well as a DNA encoding for a reverse transcriptase of LINE retrotransposons (RT LINE) [34], [36], [37]. Furthermore, the *in vitro* methylation of tRNA^Asp^ was also demonstrated for EhMeth [38], [39].

In this study, the crystal structure of the binary complex comprising EhMeth and AdoHcy is presented at a resolution of 2.15 Å. In contrast to the human DNMT2 structure, all structural elements of EhMeth could be resolved, including the active site loop. In addition, tRNA binding studies suggest that EhMeth interacts with a RNA substrate only containing 17 nucleotides of the tRNA anticodon stem-loop (including the substrate cytosine C38), but requires a full-length tRNA for stable complex formation.

## Results

### Crystallization and structure solution

EhMeth crystallized in multiple conditions, however, best diffracting crystals were obtained in conditions containing PEG 8000 and ammonium sulfate. The crystals appeared infrequently, as long rods, after two to three weeks and grew to a size of approximately 500×90×90 µm. Crystals belong to space group P4_3_2_1_2 and contain one molecule per asymmetric unit. The structure was solved by molecular replacement using the human DNMT2 structure as a search model (PDB-ID: 1G55) [19]. The structure was refined at a resolution of 2.15 Å giving rise to electron density of high quality (Table 1). Out of 322 residues, 320 are defined in the electron density, two residues at the N-terminus were not properly resolved probably due to increased flexibility in this area. All residues lie within allowed Ramachandran areas, with the exception of the active site residue Ser303. Notably, Ser303 displays very well defined electron density and its unfavorable conformation is held in position by an interaction to various surrounding residues. Its carbonyl oxygen is bound to Arg165 and forms water-bridged interactions to Glu124 and Arg167, the amide nitrogen interacts with the carbonyl oxygen of Cys299 and Leu300, and the side-chain hydroxyl group interacts with the main-chain atoms of Leu300 and Val304 as well as with the imidazole ring of His278. After structure refinement, several tetrahedral difference map peaks could be observed in the electron density that either resembled phosphate or sulfate ions, all of which were modeled as sulfates due to the presence of sulfate in the crystallization condition.

**Table 1:** Data Collection and Refinement Statistics.

Data collection		Refinement	
**Wavelength (Å)**	0.9181	Resolution limits (Å)	46.46-2.15
***Cell dimensions (Å)***		No. of used reflections	18527
**a**	47.02	No. of protein atoms	2621
**b**	47.02	No. of ligand atoms	43
**c**	303.63	No of water atoms	222
**α**	90.0	R-factor (%)	21.83
**β**	90.0	R_free_ (%)	26.71
**γ**	90.0		
**Space group**	P4_3_2_1_2	*Ramachandran plot statistics*	
**Resolution range (Å)**	50.0– 2.15	Most favourable region (%)	97.2
**No. of reflections**	50783 (7357)	Generously allowed regions (%)	2.5
**Average I/σ**	10.99 (2.46)	Disallowed regions (%)	0.3
**Completeness (%)**	99.1 (99.9)	*r.m.s. deviations from ideal values*	
**Redundancy**	3.48 (3.58)	Bond distance (Å)	0.002
**R_merge_ (%)**	9.9 (51.5)	Angles (°)	0.614

Values in parentheses indicate the specific values in the highest resolution shell.

R = Σ(||F_obs_|−scale |F_model_||)/Σ(|F_obs_|).

R_merge_ = Σ_hkl_Σ*_i_*|I*_i_*(hkl)−<I*_i_*(hkl)>|/Σ_hkl_Σ*_i_*<I*_i_*(hkl)>, where the sum *i* is over all separate measurements of the unique reflection hkl.

R_free_ as R-factor, but summed over a 5.93% test set of reflections.

### Overall structure

Overall, EhMeth displays a high structural homology to human DNMT2 and to the *H. haemolyticus* cytosine 5-methyltransferase (M.*Hha*I) (Table 2), however, the structure shows additional features that could not be resolved in the DNMT2 crystal structure. EhMeth consists of a large domain and a small domain that are connected by a hinge region. The large domain harbors the N- and C-terminus of the protein and consists of a 6-stranded β-sheet, which is surrounded by three α-helices on each side. In contrast to human DNMT2, the crystal structure of EhMeth also reveals the active site loop (residues 80–100), which adopts an α-helical conformation. In M.*Hha*I, this loop has been shown to interact with DNA [8]. However, the conformation of the active site loop in EhMeth is stabilized by crystal packing contacts to neighboring molecules. Therefore it is conceivable that it adopts a different conformation in a complex with a nucleic acid substrate. Since the sequence in the active site loop is almost identical in DNMT2, it is tempting to speculate that the active site loop of DNMT2 and EhMeth are both flexible to accommodate for either DNA or tRNA substrate binding.

**Table 2:** Similarity of EhMeth to selected methyltransferases.

organism	PDB-ID	sequence identity (%)	r.m.s.d. (Å) (number of matched residues)
***Entamoeba histolytica*** ** EhMeth (this study)**	3QV2	100	0
***Homo sapiens*** ** DNMT2**	1G55	27.6	1.03 (260)
***Haemophilus haemolyticus*** ** M.** ***Hha*** **I (closed conformation)**	1MHT	20.5	1.66 (237)
***Haemophilus haemolyticus*** ** M.** ***Hha*** **I (open conformation)**	1HMY	20.5	1.59 (224)

**Figure 1:**
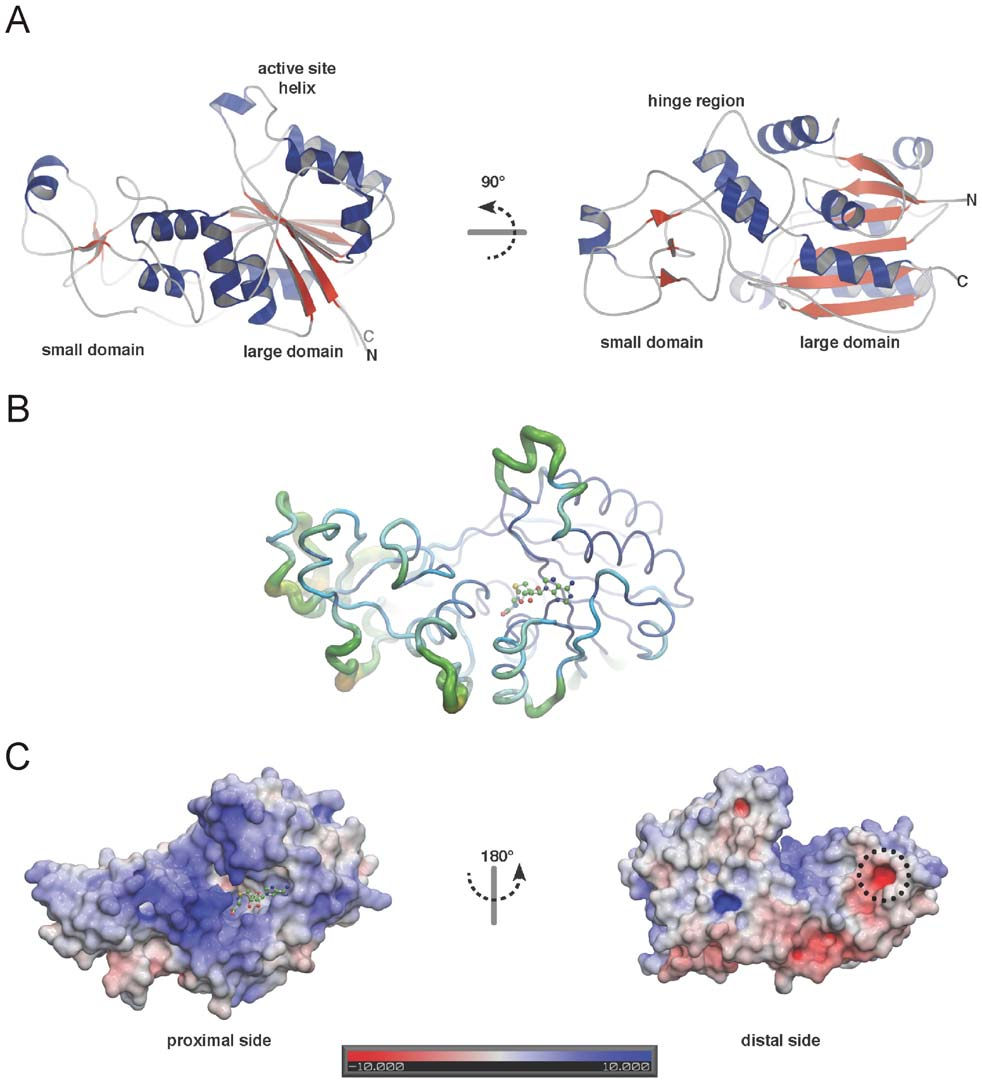
The crystal structure of EhMeth. (A) Secondary structure representation: α-helices are shown in blue, β-strands in red while loop regions are depicted in grey. Clearly the active site helix is protruding from the large domain. (B) The main-chain of EhMeth is shown in relation to its B-factor. Increased B-factors indicate a higher flexibility of the respective area of the structure, which can be observed in the active site helix and some loop areas in the small domain. (C) Surface charge representation: the proximal side of EhMeth is predominantly positively charged (blue), while the distal side of EhMeth mainly displays neutral and negative surface charges (grey, red). On the positively charged surface – the putative DNA/RNA binding area – also the cofactor-binding pocket can be seen (AdoHcy is shown in green). A dashed circle highlights a highly negatively charged pocket on the distal side of EhMeth.

Two α-helices connected by a loop form the hinge region that link the large domain to one end of the small domain (Figure S1 and S3). At the other end the small domain is connected to the large domain by a long loop (residues 175–195) spanning almost the whole length of the structure. The small domain itself consists of a short 3-stranded β-propeller and a single α-helix (Figure 1a). Overall the large domain is relatively rigid while the small domain shows a higher flexibility indicated by increased B-factors (Figure 1b). Calculation of the solvent accessible surface charge reveals one side that displays a neutral to slightly acidic charge (distal), while the other side of EhMeth is highly positively charged (proximal) (Figure 1c). Moreover, on the positively charged side of EhMeth a large pocket can be observed. In this pocket, a strong difference map peak clearly resembling an AdoHcy-molecule was visible after modeling of the protein residues. Hence, an AdoHcy-molecule was included in the cofactor-binding pocket in final refinement steps.

### The S-Adenosyl-Homocysteine binding site

**Figure 2:**
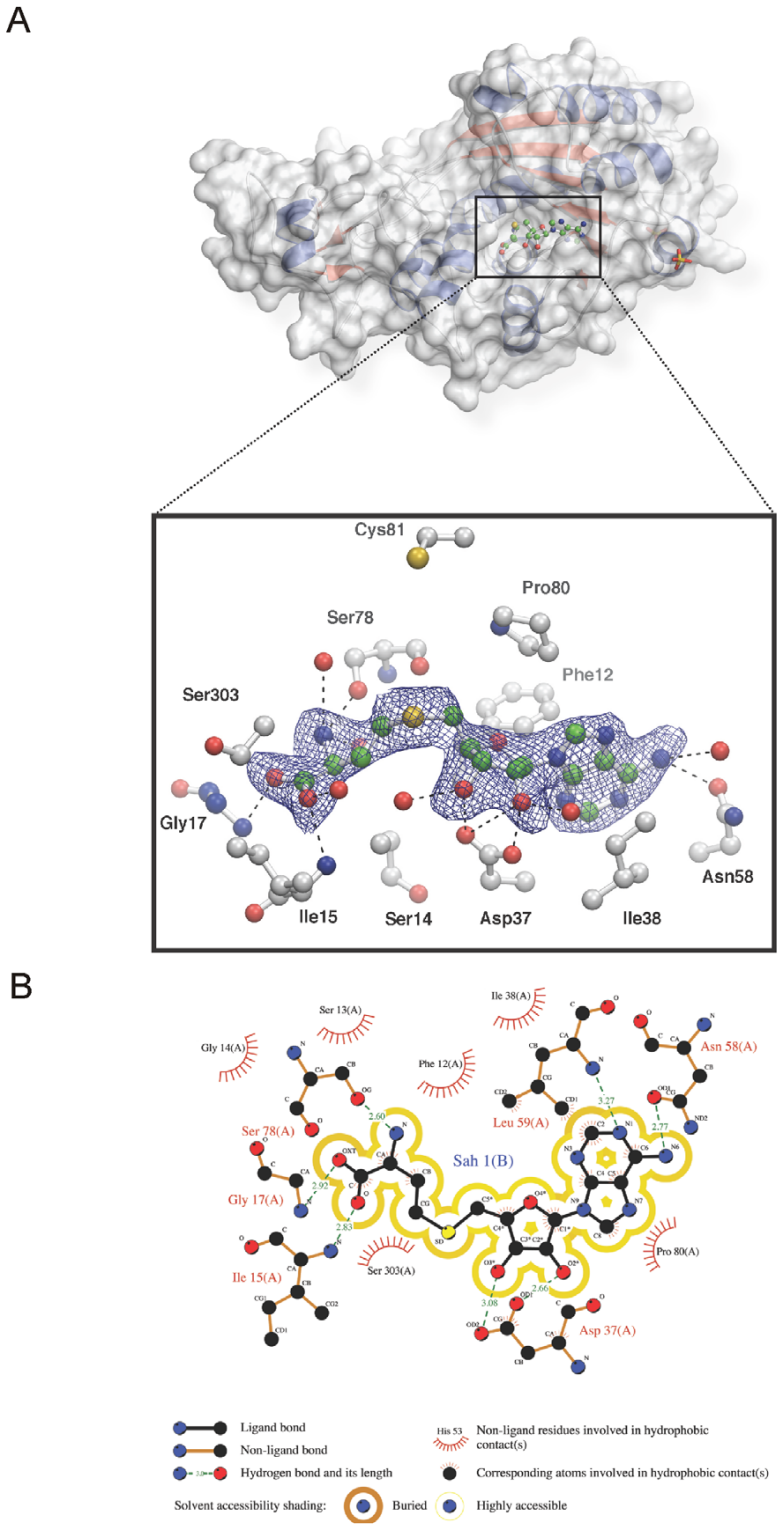
The cofactor-binding pocket. (A) A surface representation of EhMeth shows the cofactor-binding pocket in the large domain of the protein. The binding of AdoHcy in the pocket is mediated by a variety of polar and non-polar interactions shown in the close-up view. A |F_O_−F_C_| simulated annealing omit map contoured at 1.5 σ (blue mesh) supports the conformation of the ligand. AdoHcy is shown in a green while the interacting residues are shown in a grey ball and stick representation. (B) A LIGPLOT diagram shows the 2-dimensional projection of the protein residues interacting with the ligand. Distances between individual interacting atoms are indicated.

The presence of AdoHcy in the active site was further supported by calculating a |F_O_−F_C_| simulated annealing omit map (Figure 2a). AdoHcy is bound by various polar and non-polar interactions: The adenine moiety forms hydrogen bonds to the side-chain of Asn58 as well as to the backbone amide of Leu59. However, it is also kept in position by an edge-to-face van der Waals interaction with Phe12, an environment that is further supported by interactions with Ile38 and Pro80. The ribose forms hydrogen bonds to Asp37, while the cysteinyl moiety contacts the main chain atoms of Ile15, Gly17 and Ser78. Here, binding is also supported by hydrophobic interactions provided by main-chain carbons from Ser13, Gly14 and Ser303 (Figures 2a & 2b).

### Model of the EhMeth DNA complex

**Figure 3:**
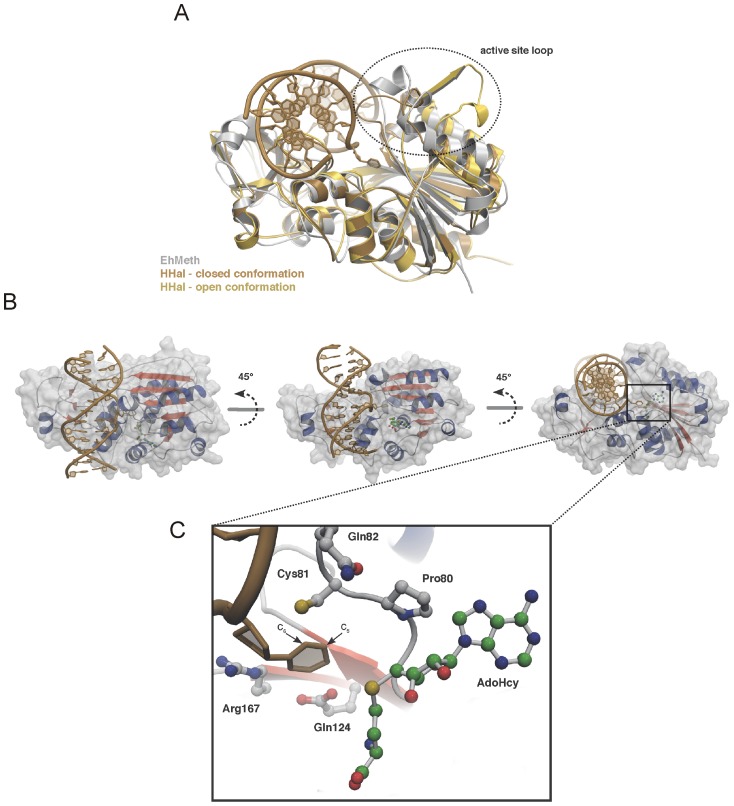
Model of the EhMeth-DNA complex. (A) Superposition of EhMeth (grey) and M.*Hha*I in closed conformation (brown) and open conformation (yellow). The superposition clearly illustrates that the active site loop of EhMeth adopts a conformation between the open and the closed conformation of M.*Hha*I. (B) Due to the high structural homology between EhMeth and M.*Hha*I a DNA-binding model could be derived from superposition with the substrate bound M.*Hha*I-structure. The M.*Hha*I-DNA neatly fits into the putative DNA-binding site of EhMeth. (C) The close-up of the active site illustrates that the flipped out cytosine is in a conformation that would allow for methyl-group transfer further supporting, that EhMeth follows the same reaction mechanism as M.*Hha*I and DNMT2.

In contrast to the human DNMT2 structure, the active site loop (residues 80–100) of EhMeth is well defined and clearly forms an α-helix. The conformation of the active-site helix is stabilized by crystal packing contacts to the long loop connecting the large and the small domain of neighboring molecules. To compare the conformation of the active site loop with similar 5-cytosine methyltransferases, EhMeth was superposed with the structure of M.*Hha*I in the closed conformation (PDB-ID: 1MHT) and M.*Hha*I in the open conformation (PDB-ID: 1HMY) [8]. The superposition illustrates how the active site loop is involved in binding the substrate DNA (Figure 3a). In the closed conformation of M.*Hha*I the active site loop is in intimate contact with the minor groove of the DNA. However, in the open conformation, it is pointing away from the DNA. Interestingly, the active site helix of EhMeth is stabilized in a conformation that lies between the open and the closed conformation of M.*Hha*I. However, the high sequence identity shared by M.*Hha*I, DNMT2 and EhMeth suggests that the active site loop in EhMeth and DNMT2 will express the same flexibility as in M.*Hha*I. This would allow it to change its position depending on substrate binding and adopt a closed conformation in presence of a substrate and an open conformation in its absence (Figure 3a).

All attempts to co-crystallize EhMeth with a double-stranded DNA (dsDNA) oligomer have failed so far. However, since EhMeth and the structure of M.*Hha*I (PDB-ID: 1MHT) can be superposed with an r.m.s.d of 1.6 Å among the C_α_-atoms (Table 2), the superposition yields a convincing model of the EhMeth-DNA complex (Figure 3b). Albeit a natural DNA-EhMeth complex would result in a better, presumably tighter, fit between the protein and the DNA, the current model does not result in any protein backbone clashes. The only clashes that can be observed are between two lysine residues (Lys236, Lys294) and a thymine nucleotide within one of the DNA strands. More than 20 residues in M.*Hha*I contribute to DNA binding. Not surprisingly, the residues involved in methyl group transfer in the active site are strictly conserved in EhMeth (Cys81, Glu124, Arg167). The superposition with M.*Hha*I DNA illustrates that these residues adopt a conformation that would allow for successful catalysis (Figure 3). Moreover, on the large domain and in the hinge region also a number of other conserved residues can be found that are involved in DNA binding in M.*Hha*I at structurally equivalent positions in EhMeth (Gln82, Ile88, Lys91, Arg99, Gln295). Presumably these residues are also involved in DNA binding of EhMeth.

**Figure 4:**
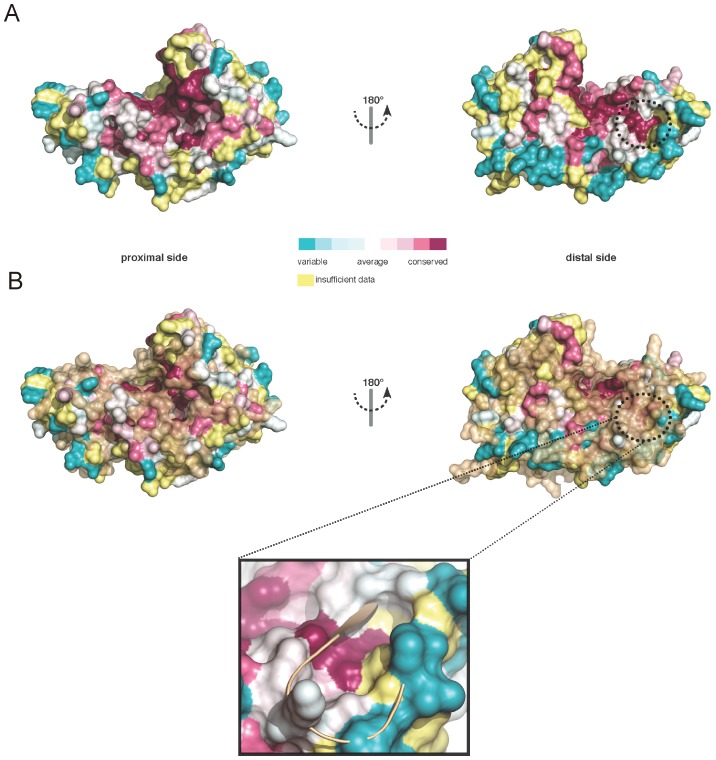
Conservation among DNMT2 enzymes. (A) Conserved residues among DNMT2 MTases were mapped to the surface of EhMeth. Purple color displays high conservation, cyan color displays high variance. Highly conserved areas can be seen in the active site, the putative DNA/tRNA binding site as well as in a highly acidic pocket on the distal site of EhMeth. (B) A superposition of the conserved areas with the surface of M.*Hha*I (closed conformation, brown) shows that only minor divergence can be observed at the proximal site while in particular the acidic pocket on the distal side is covered – that is not existent in M.*Hha*I. The close-up illustrates that the acidic pocket is occupied by strand β10.

However, at the small domain only Arg226 is conserved in EhMeth. Interestingly, this residue cannot be found at a structurally equivalent position but is shifted by approximately 10 Å more to the proximal side of the protein. It is interesting to note that this shift is not only restricted to Arg226, but all surrounding residues are shifted to the proximal side of EhMeth. Intriguingly, this causes the formation of an acidic pocket (in proximity to Asp218) on the surface of EhMeth at the position that is occupied by strand β10 in M.*Hha*I (Figures 1, 4 & S2).

An alignment of selected DNMT2 sequences from several distinct eukaryotic kingdoms (Figure S1) was mapped onto the surface of EhMeth, showing that this acidic pocket is strictly conserved. Moreover, structural comparison with M.*Hha*I shows that this pocket cannot be found in the bacterial MTase arguing for a DNMT2 specific feature (Figure 4 & S2).

### tRNA and DNA binding of EhMeth

**Figure 5:**
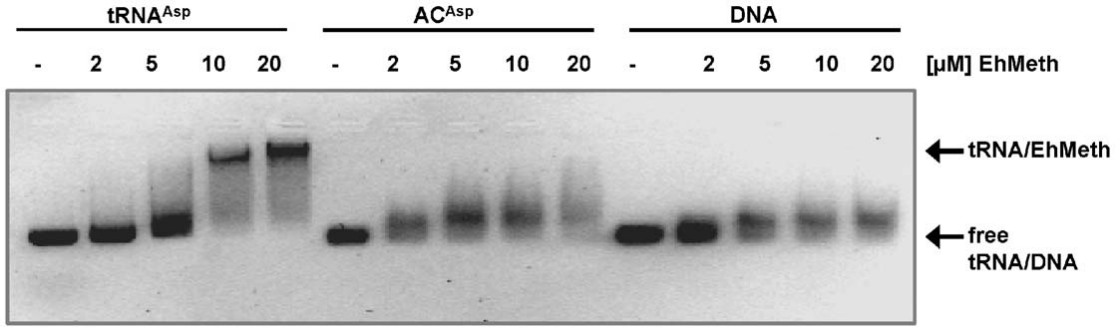
Electrophoretic mobility shift assay with EhMeth. Different nucleic acid substrates (full length tRNA, 17 nucleotide anticodon stem-loop and DNA) were subjected to increasing concentrations of EhMeth. Arrows highlight the position of free RNA/DNA bands in comparison to the tRNA bound to EhMeth. In contrast to full length tRNA, which is clearly shifted to a distinct band, the anticodon stem loop as well as DNA are only slightly shifted and smearing upon addition of EhMeth.

In order to analyze whether the anticodon stem loop of tRNA^Asp^ is sufficient for binding to EhMeth or whether the complete tRNA molecule is required, mobility shift assays were performed. Indeed only in presence of the full-length tRNA^Asp^ substrate a well-defined band shift representing the EhMeth-tRNA complex can be observed (Figure 5). In contrast, EhMeth and the anticodon stem-loop seem to interact only in a very weak or transient fashion, since the free RNA band is fading with increasing concentrations of EhMeth and is not shifted to a clear-defined band as detected for full-length tRNA. In conclusion, EhMeth interacts with the anticodon stem-loop but for tight binding to EhMeth the full-length tRNA is required.

Additionally, electrophoretic mobility shift assays were conducted employing the identical DNA substrate as used for co-crystallization of M.*Hha*I (PDB-ID: 1MHT). Similar to the anticodon stem-loop, the DNA is not completely shifted but results in an intermediate band, which is fading with increasing concentrations of EhMeth. The mobility shift assays suggest that the duplex DNA and the anticodon stem-loop bind to EhMeth in a very similar way, since they both result in an identical band shift pattern. In contrast, the full length tRNA is shifted substantially, resulting in a distinct band in the native gel, which implicates a different and presumably tighter binding mode.

## Discussion

In previous studies it was demonstrated that the only DNA MTase in *E. histolytica*, EhMeth, belongs to the DNMT2-family and has dual substrate specificity being able to methylate DNA as well as C38 of tRNA^Asp^ [34], [38], [39]. The three-dimensional structure of EhMeth shows a high structural homology to the bacterial DNA MTase M.*Hha*I and to human DNMT2, which could make the design of a species-specific inhibitor a challenging task. The structural homology manifests itself not only in the general fold, but is also present in all elements required for methyl-group transfer and in many characteristics required for DNA binding (Figures 3 & S1). This homology suggests that EhMeth utilizes the same reaction mechanism for methyl-group transfer as shown for DNMT2 and M.*Hha*I [9], [30], [34]. The observed AdoHcy conformation as well as the DNA-binding model presented in this study, further supports this hypothesis.

However, the high similarity between bacterial DNA MTases and DNMT2 enzymes raises the question what structural differences exist, that prevents the bacterial enzymes from methylating tRNA and allows DNMT2 enzymes to select only for specific tRNAs.

While the large domain, required for methyl-group transfer, is relatively invariant among MTases, the small domain has been associated with altered substrate specificity [3]. In line with this the small domains of M.*Hha*I, DNMT2 and EhMeth display indeed notable differences to each other. While all small domains of the three MTases share the short, central β-propeller motif, the surrounding elements relevant for substrate recognition are different. The small domain of M.*Hha*I is almost exclusively β-stranded, whereas the small domain in DNMT2 and EhMeth is predominantly α-helical. In spite of this difference, a single residue located in strand β10 in M.*Hha*I that was shown to interact with DNA, is also conserved in DNMT2 and EhMeth [8]. However, the residues forming strand β10 in M.*Hha*I are shifted more to the proximal side in DNMT2 and EhMeth and the conserved residue (Arg226 in EhMeth) cannot be found at a structurally equivalent position (Figures 4 & S2). Notably the relocation of strand β10 is accompanied with the formation of an acidic pocket at a Φ-D-I-V motif (where Φ is a hydrophobic residue) that is strictly conserved in DNMT2-MTases (Figures 4a & S1). Size and shape of the acidic pocket suggest that it has ideal proportions to accommodate the base of a nucleotide, thus could play a role in substrate binding. At first glance this may appear to be a subtle difference but due to the strong conservation this clearly discriminates DNMT2 enzymes from bacterial MTases. Furthermore, this conserved change is one of the few highly charged positions on the distal side of EhMeth (Figures 1 & S2), and in direct proximity to an area that was shown to be important for substrate binding in M.*Hha*I. Furthermore, no substantial difference between EhMeth and the bacterial MTase can be observed at the proximal side of the enzymes (Figure 4b).

One explanation for the conservation of the acidic pocket could be an altered sequence specificity of DNMT2 enzymes. EhMeth was shown to methylate specific sequences in S/MAR regions and LINE retrotransposons [36], [37]. This demonstrates target selectivity among DNA sequences, and indicates a role of EhMeth in the regulation of retrotransposons among *Entamoeba* species. However, the conservation of the acidic pocket could also be explained by the ability of DNMT2 enzymes to recognize specific tRNAs. In support of the latter hypothesis, the conserved Arg226 as well as the Φ-D-I-V motif are in close proximity to the target recognition domain (TRD) harboring the CFT sequence motif (CFTxxYxxY, where x is any amino acid), which is unique to DNMT2 enzymes [19].

To decipher the function of DNMT2-MTases, another important question is to understand what distinguishes the target tRNAs from non-cognate tRNAs. Crystal structures of tRNA-modifying enzymes bound to tRNA reveal that tRNA recognition often not only depends on the nucleotide to be modified, but also requires interactions with additional parts of the tRNA [40], [41]. For the bacterial tRNA-MTase TrmA, it was suggested that in addition to the T-loop the D-loop interacts with the protein [42]. Similarly, the eukaryotic tRNA-MTase Trm5 interacts not only with the anticodon loop harboring the substrate nucleotide G37, but also contacts the D- and T-loop [43]. Both loops have been reported to be more stable in fully modified and mature tRNA molecules [44]. As a consequence, Trm5 specifically selects and modifies tRNA species, which adopt the correct conformation and thus may also serve as a sensor for proper folding and accurate tRNA maturation [43]. TiaS in contrast recognizes the acceptor stem of the tRNA in addition to specific interactions with the substrate nucleotide C34 located in the anticodon loop [45].

Clearly, DNMT2 enzymes such as EhMeth do not discriminate against the anticodon sequence of the tRNA itself, since the enzyme was shown to methylate tRNA^Asp^, tRNA^Gly^ as well as tRNA^Val^ [23], [28], [29]. A singular discriminating feature is also unlikely to be found in the anticodon stem loop, since the mobility shift assays clearly show that EhMeth depends on additional parts of the L-shaped tRNA to recognize its substrate in a stable manner (Figure 5). Therefore, the discriminating property of the target tRNAs has to be found in other areas of the tRNAs. Indeed, alignments of target tRNA^Asp^, tRNA^Gly^ and tRNA^Val^ from different organisms reveal a common sequence motif (UAGUNΨ) located at the 5′ end of the D-stem (Figure S4). Hence, it is tempting to speculate that the D-stem plays an important role in substrate recognition. However, also tRNA^His^ and tRNA^Glu^ comprise this motif in their D-stem indicating that additional criteria need to be fulfilled for substrate discrimination. Most obvious is the presence of a guanosine at position 39 in tRNA^Asp^, tRNA^Gly^ and tRNA^Val^. Interestingly, these three tRNAs share a cytosine at the third position of the anticodon sequence.

To understand the dual substrate acceptance of DNMT2 MTases and the specificity for three tRNAs one inevitably needs to analyze a DNMT2-tRNA complex structure. The co-crystal structure of the methyltransferase with its substrate tRNA will provide profound insights into the protein-tRNA interactions involved in this fundamental process.

### Conclusions

The crystal structure of the cytosine-5-methyltransferase EhMeth (DNMT2) in complex with AdoHcy shows high structural similarity to the DNA MTase M.*Hha*I and to human DNMT2. Mobility shift assays show the requirement of the full-length tRNA for stable complex formation *in vitro*. We hypothesize that a conserved region on the distal side of EhMeth could be involved in tRNA binding.

## Materials and Methods

### Protein Purification and Crystallization

EhMeth was cloned into the pGEX6-P3 vector (GE Healthcare); protein expression was conducted in *Escherichia coli* BL21(DE3) in TB-medium at 16°C for 18 h. The GST-fusion protein was purified using a GSH-sepharose (0.1 M Tris/HCl pH 7.25, 0.25 M NaCl, 0.001 M DTT) following the manufacturers' instructions. In order to reduce the contamination with nucleic acids an initial wash step including 1 M LiCl was performed. Subsequently the eluate was incubated with precission protease (1∶100 w/w) for 18 h at 4°C. In order to remove cleaved GST as well as contaminating nucleic acids, a heparin-sepharose purification step was included (0.1 M Tris/HCl pH 7.25, 0.1 M NaCl–2 M NaCl, 0.001 M DTT). EhMeth was further purified by Superdex 75 size exclusion chromatography (0.01 M Tris/HCl pH 7.25, 0.1 M NaCl). Purified EhMeth was concentrated to 4.5 mg/ml and stored at −80°C until further usage. Protein concentration was determined by Bradford assay. Crystals were grown at 20°C in sitting drop vapour diffusion plates combining equal volumes of precipitant (0.1 M MES pH 6.5, 22% PEG 8000 (w/v), 0.2 M (NH_4_)_2_SO_4_) and the protein solution. Additionally, the protein was supplemented with a 150× molar excess of AdoHcy and an equimolar amount of a synthetic RNA-oligo (5′-CCCGCCUGUCACGCGGG-3′) for co-crystallization attempts. Crystals were cryo-protected by soaking in precipitant solution containing additionally 12% (v/v) 2,3-butantediol and flash frozen in liquid nitrogen prior to data collection.

### Data collection and processing

X-ray data collection was performed at 100 K; diffraction images were collected at beamline 14.1 at BESSY, Berlin. Diffraction images were indexed, integrated and scaled using the XDS-package [46].

### Molecular replacement and structure refinement

The structure was solved by molecular replacement (PHASER) using the human DNMT2 structure as a search model (PDB-ID: 1G55) [19], [47]. Model building was conducted in COOT [48]. Coordinates were refined to reasonable stereochemistry at a resolution of 2.15 Å using PHENIX (Table 1) [49]. Alternating steps of refinement and structure adjustments were performed until the R-values converged. The structure was validated using MOLPROBITY [50]. Simulated annealing omit maps were calculated in CNS [51].

### Structure and sequence comparison

The calculation of r.m.s.d.'s has been performed with the program LSQMAN [52]. A distance cut-off of 3.5 Å was applied in all brute-force alignments. Sequence identity calculations were performed using LALIGN calculating a global alignment of two sequences [53]. Protein sequences were aligned using CLUSTALW [54]. Alignment graphics were colored using ESPript [55]. Conserved residues were mapped to the protein surface using the CONSURF web-server [56], [57], [58]. TRNA sequence alignments were derived from tRNAdb [59].

### Molecular visualization

Molecular images were generated in Pymol [60]. Electrostatic surface potentials were calculated using APBS [61], [62], [63]. 2D plots of the ligands were generated using LIGPLOT [64].

### *In vitro* Transcription and purification of full length tRNA

The DNA template for tRNA^Asp^ was cloned in pRAV23 vector and was amplified in *E. coli* XL1-blue cells. The plasmid was extracted and purified via anion-exchange chromatography using Q-sepharose beads. The purified DNA template was concentrated employing ethanol precipitation and subjected to HindIII digestion in order to yield a run-off transcript template. To remove potential RNAse contaminations, Proteinase K was added and subsequently denatured by heating to 95°C. 1–1.5 mg DNA template was employed in an *in vitro* transcription approach containing T7 Polymerase and each 40 mM rNTPs in 1× HT buffer (30 mM HEPES pH 8.0, 25 mM MgCl_2_, 10 mM DTT, 2 mM Spermidine, 0.01% Triton X-100). After incubation at 37°C for 16 h, the transcript was applied to ethanol precipitation and cleavage of the 3′ ribozyme GlmS from the tRNA was induced by the addition of 5 mM GlcN6P. To separately elute the tRNA and the ribozyme anion exchange chromatography including a very shallow salt gradient was performed. The elution fractions were analysed on a denaturing urea gel for the presence and purity of the tRNA.

### Electrophoretic mobility shift assays

EhMeth was incubated with tRNA or DNA substrates for 25 min at room temperature in reaction buffer containing 50 mM HEPES pH 8.0, 50 mM KCl, 10 mM MgCl_2_, 1 mM EDTA, 0.5% (w/v) Glycerol, 0.1 mg/ml BSA and 1 mM DTT. Nucleic acids were applied in a final concentration of 2 µM with increasing concentrations of EhMeth ranging from 2–20 µM. Native loading dye containing glycerol and Orange G was added and samples were loaded on a 1% agarose gel supplemented with GelRed. Electrophoresis was performed at 111 V for 20 min on ice using pre-cooled 1× TAE running buffer. The gel was subsequently exposed to UV-light to visualize nucleic acids. Full length tRNA was generated as described in 4.6., whereas the anticodon stem loop (5′-CCCGCCUGUCACGCGGG-3′) and DNA (5′-GATAGCGCTATC-3′ duplexed with 5′-TGATAGCGCTATC-3′ in order to form a duplex comprising a single nucleotide 5′-overhang) were purchased from IBA and Sigma Aldrich, respectively.

### Accession numbers

Coordinates and structure factors for the EhMeth structure have been deposited in the Protein Data Bank (http://www.rcsb.org/pdb/home/home.do) with the entry code 3QV2. The Protein Data Bank accession numbers for the proteins discussed in the paper are as follow: DNMT2 (1G55), M.*Hha*I (1MHT and 1HMY).

## Supporting Information

Figure S1 **Sequence alignment of DNMT2 enzymes.** Amino acid sequence alignment of DNMT2 enzymes from selected organisms, structural elements found in EhMeth are indicated above. The same sequences were used to determine conserved regions in DNMT2 enzymes depicted in Figure 4. The ϕDIV-motif is indicated by a black box.
(PDF)

Figure S2 **Superposition of EhMeth and M.** ***HHa*** **I.** (A) EhMeth is shown in surface charge representation – M.*HHa*I is shown as brown ribbons. Clearly strand β10 occupies the position of the acidic pocket in EhMeth, indicated by a dotted circle. (B) Relocation of M.*HHa*I strand β10 – the conserved residues in EhMeth and DNMT2 cannot be found in a structurally equivalent position but have been relocated further to the proximal side of the protein.
(TIFF)

Figure S3 **Ribbon representation of EhMeth.** The naming of the individual secondary structure elements is consistent with the sequence alignment shown in Figure S1.
(TIF)

Figure S4 **Sequence alignment of target tRNA sequences.** The nucleotide sequence alignment was derived from the tRNA database tRNAdb (http://trnadb.bioinf.uni-leipzig.de/) using *Arabidopsis thaliana*, *Drosophila melanogaster* and *Homo sapiens* tRNA sequences comprising the anticodons GTC, GCC and AAC (encoding for Asp, Gly and Val, respectively). These anticodons have been previously shown to be modified by DNMT2 enzymes from the above mentioned organisms.
(PDF)
