# Supplementary material for: Structure Analysis of Entamoeba histolytica DNMT2 (EhMeth)
Source: PLoS One. 2012 Jun 21;7(6):e38728. doi: 10.1371/journal.pone.0038728 (PMC3380923; doi:10.1371/journal.pone.0038728)
Supplement: Figure S1 — Sequence alignment of DNMT2 enzymes. Amino acid sequence alignment of DNMT2 enzymes from selected organisms, structural elements found in EhMeth are indicated above. The same sequences were used to determine conserved regions in DNMT2 enzymes depicted in Figure 4. The ϕDIV-motif is indicated by a black box. (PDF) [file pone.0038728.s001.pdf]

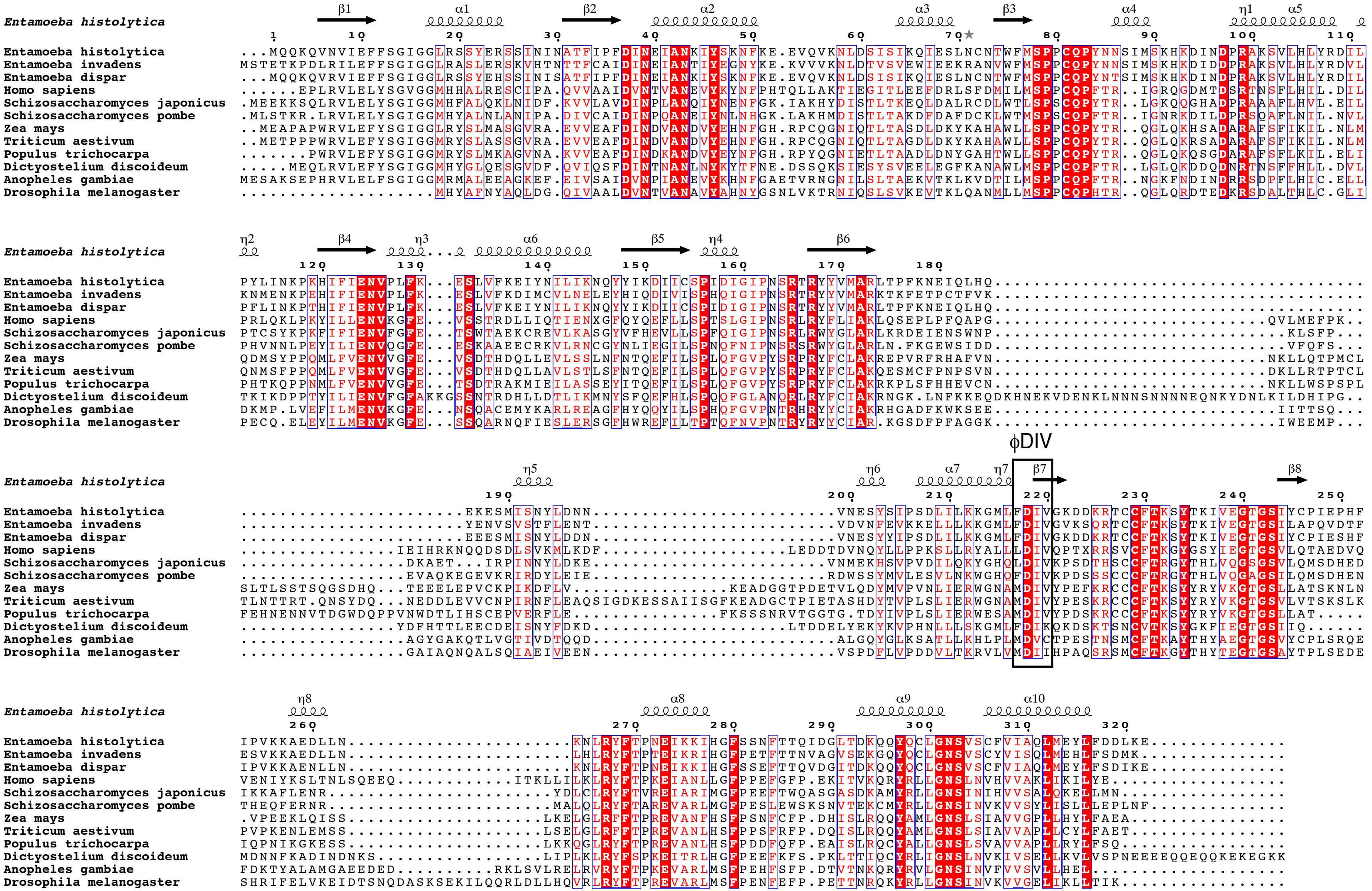
Entamoeba histolytica
β1
α1
β2
α2
α3
β3
α4
η1
α5
Entamoeba histolytica
Entamoeba invadens
Entamoeba dispar
Homo sapiens
Schizosaccharomyces japonicus
Schizosaccharomyces pombe
Zea mays
Triticum aestivum
Populus trichocarpa
Dictyostelium discoideum
Anopheles gambiae
Drosophila melanogaster
η2
β4
η3
α6
β5
η4
β6
ϕDIV
η5
η6
α7
η7
β7
β8
η8
α8
α9
α10
