# Supplementary figures and images for: Structure Analysis of Entamoeba histolytica DNMT2 (EhMeth)
Source: PLoS One. 2012 Jun 21;7(6):e38728. doi: 10.1371/journal.pone.0038728 (PMC3380923; doi:10.1371/journal.pone.0038728)

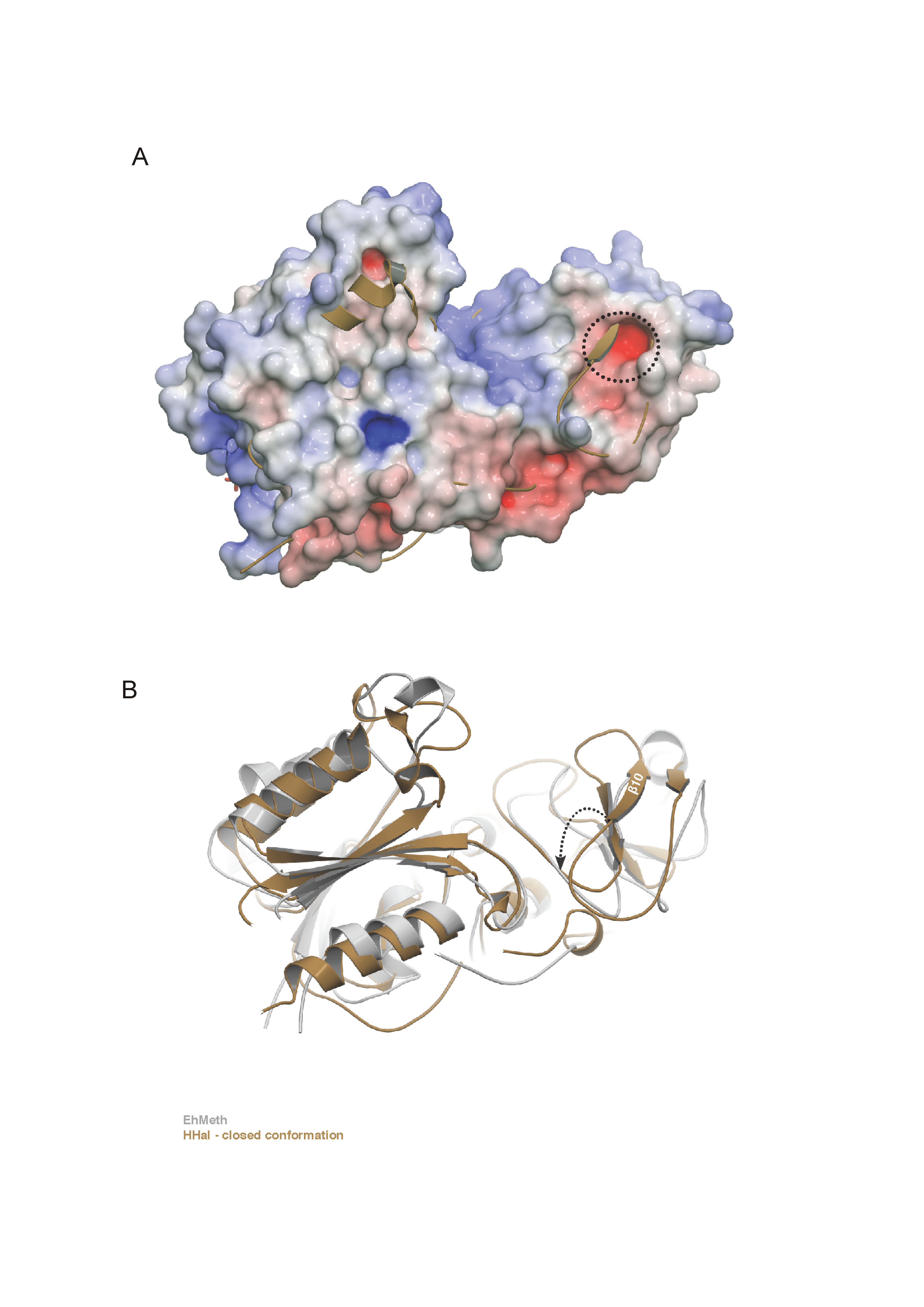

Supplement: Figure S2 — Superposition of EhMeth and M. HHa I. (A) EhMeth is shown in surface charge representation – M.HHaI is shown as brown ribbons. Clearly strand β10 occupies the position of the acidic pocket in EhMeth, indicated by a dotted circle. (B) Relocation of M.HHaI strand β10 – the conserved residues in EhMeth and DNMT2 cannot be found in a structurally equivalent position but have been relocated further to the proximal side of the protein. (TIFF) [file pone.0038728.s002.tiff]

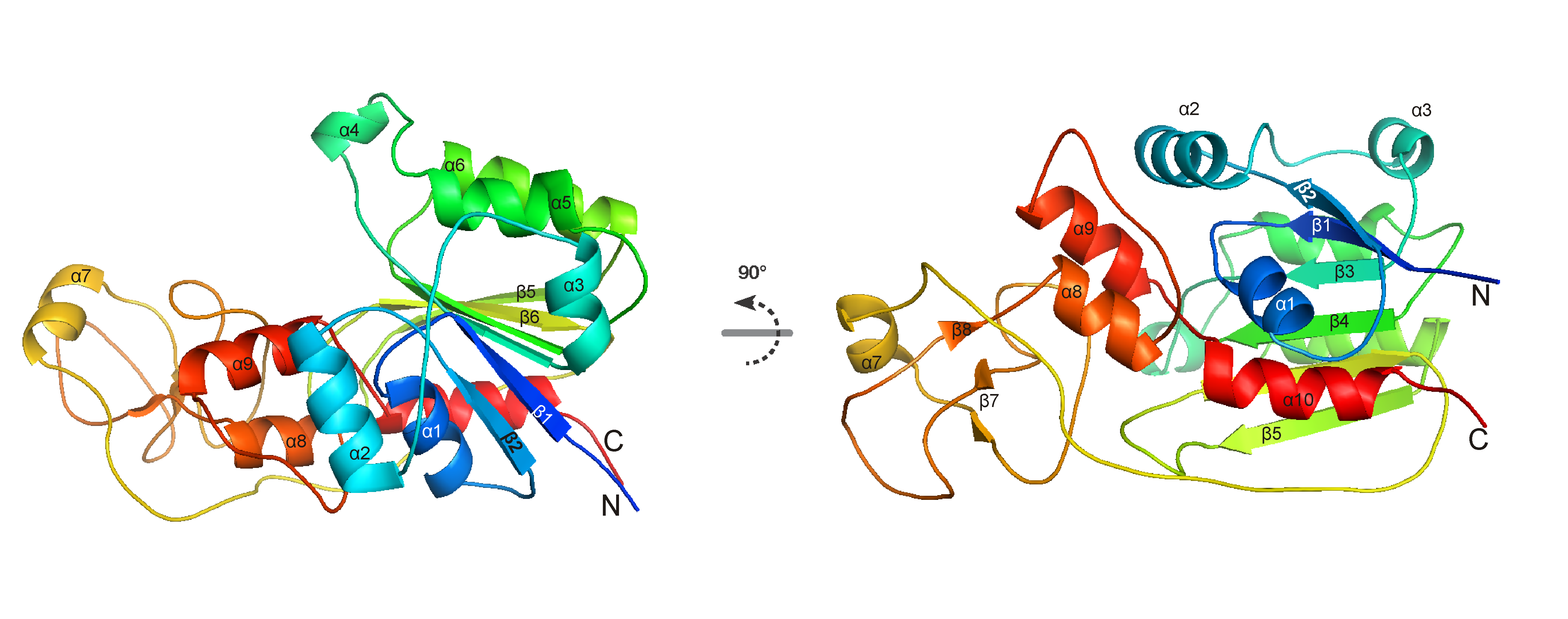

Supplement: Figure S3 — Ribbon representation of EhMeth. The naming of the individual secondary structure elements is consistent with the sequence alignment shown in Figure S1. (TIF) [file pone.0038728.s003.tif]
