# Supplementary material for: Structure Analysis of Entamoeba histolytica DNMT2 (EhMeth)
Source: PLoS One. 2012 Jun 21;7(6):e38728. doi: 10.1371/journal.pone.0038728 (PMC3380923; doi:10.1371/journal.pone.0038728)
Supplement: Figure S4 — Sequence alignment of target tRNA sequences. The nucleotide sequence alignment was derived from the tRNA database tRNAdb (http://trnadb.bioinf.uni-leipzig.de/) using Arabidopsis thaliana, Drosophila melanogaster and Homo sapiens tRNA sequences comprising the anticodons GTC, GCC and AAC (encoding for Asp, Gly and Val, respectively). These anticodons have been previously shown to be modified by DNMT2 enzymes from the above mentioned organisms. (PDF) [file pone.0038728.s004.pdf]

```
                                    1        10        20         30        40         50        60
tdbD00000964|Drosophila_melano  .GCATCGGTGGTTCAGT.GGTAG..AATGCTCGCCTGCCACGCGGGCGG.CCCGGGTTCGATTCCCGGCC
tdbD00005350|Drosophila_melano  .GCATCGGTGGTTCAGT.GGTAG..AATGCTCGCCTGCCACGCGGGCGG.CCCGGGTTCGATTCCCGGCC
tdbD00000965|Drosophila_melano  .GCATCGGTGGTTCAGT.GGTAG..AATGCTCGCCTGCCACGCGGGCGG.CCCGGGTTCGATTCCCGACC
tdbD00005349|Drosophila_melano  .GCATCGGTGGTTCAGT.GGTAG..AATGCTCGCCTGCCACGCGGGCGG.CCTGGGTTCGATTCCCGGCC
tdbD00000970|Homo_sapiens|9606  .GCATTGGTGGTTCAGT.GGTAG..AATTCTCGCCTGCCACGCGGGAGG.CCCGGGTTCGATTCCCGGCC
tdbD00005318|Homo_sapiens|9606  .GCATTGGTGGTTCAGT.GGTAG..AATTCTCGCCTGCCACGCGGGAGG.CCCGGGTTCGATTCCCGGCC
tdbD00005317|Homo_sapiens|9606  .GCATTGGTGGTTCAGT.GGTAG..AATTCTCGCCTGCCACGCGGGAGG.CCCGGGTTTGATTCCCGGCC
tdbD00005316|Homo_sapiens|9606  .GCATAGGTGGTTCAGT.GGTAG..AATTCTTGCCTGCCACGCAGGAGG.CCCAGGTTTGATTCCTGGCC
tdbD00005319|Homo_sapiens|9606  .GCATGGGTGGTTCAGT.GGTAG..AATTCTCGCCTGCCACGCGGGAGG.CCCGGGTTCGATTCCCGGCC
tdbD00000955|Arabidopsis_thali  .GCACCAGTGGTCTAGT.GGTAG..AATAGTACCCTGCCACGGTACAGA.CCCGGGTTCAATTCCCGGCT
tdbD00005328|Arabidopsis_thali  .GCACCAGTGGTCTAGT.GGTAG..AATAGTACCCTGCCACGGTACAGA.CCCGGGTTCAATTCCCGGCT
tdbD00005327|Arabidopsis_thali  .GCACCAGTGGTCTAGT.GGTAG..AATAGTACTCTGCCACGGTACAGA.CCCGGGTTCGATTCCCGGCT
tdbD00005330|Arabidopsis_thali  .GCACCAGTGGTCTAGT.GGTAG..AATAGTACCCTGCCACGGTACAGA.CCCGGGTTCGATTCCCGGCT
tdbD00005324|Arabidopsis_thali  .TAACCAGTGGTCTAGT.GGTAG..AATAGTACCCTGCCACGGTACAGA.CCCGGGTTCGATTCCCGGCT
tdbD00005326|Arabidopsis_thali  .GCACCAGTGGTCTAGT.GGCAT..GATAGTACCCTGCCACGGTACATA.CCCGGGTTCAATTCCCGGCT
tdbD00005329|Arabidopsis_thali  .GCACCAGTGGTCTAGT.GGCAT..GATAGTACCCTGCCACGGTACAGA.CCCGGGTTCAATTCCCGGCT
tdbD00000508|Drosophila_melano  .TCCTCGATAGTATAGT.GGTTA.GTATCCCCGCCTGTCACGCGGGAGA.CCGGGGTTCAATTCCCCGTC
tdbD00004470|Drosophila_melano  .TCCTCGATAGTATAGT.GGTTA.GTATCCCCGCCTGTCACGCGGGAGA.CCGGGGTTCAATTCCCCGTC
tdbD00004469|Drosophila_melano  .TCCTCGATAGTATAGT.GGTTA.GTATCCCCGCCTGTCACGCGGGAGA.CCGGGGTTCAATTCCCCGTC
tdbD00004468|Drosophila_melano  .CCTCCGATAGTATAGT.GGTTAAGTATCCCCGCCTGTCACGCGGGAGA.CCGGGGTTCAATTCCCCGTC
tdbD00004458|Homo_sapiens|9606  .TCCTCGTTAGTATAGT.GGTTA.GTATCCCCGCCTGTCACGCGGGAGA.CCGGGGTTCAATTCCCCGAC
tdbD00004454|Homo_sapiens|9606  .TCCTCATCAGTATAGT.GGTGA.GTATCCCCGCCTGTCACGCGGGAGA.CTGGGGTTCGATTCCCCTGAG
tdbD00004457|Homo_sapiens|9606  .TCCTCGTTAGTATAGT.GGTGA.GTATCCCCGCCTGTCACGCGGGAGA.CCGGGGTTCGATTCCCCGAC
tdbD00000510|Mus_musculus|1009  .TCCTCGTTAGTATAGT.GGTGA.GTATCCCCGCCTGTCACGCGGGAGA.CCGGGGTTCGATTCCCCGAC
tdbD00004455|Homo_sapiens|9606  .TCCTCGTTAGTATGGT.GGTGA.GTATCCCTGCCTGTCACGCGGGAGA.CCGGGGTTCGATTCCCCAAC
tdbD00004456|Homo_sapiens|9606  .TCCTCGTTAGTATAGT.GGTGA.GTGTCCCCGTCTGTCACGCGGGAGA.CCGGGGTTCGATTCCCCGAC
tdbD00004453|Homo_sapiens|9606  .TACTCGTTAGTATAGT.GGTGC.GTATCCCCGTCTGTCACGCGGGAGA.GCGGGGTTCGCTCTCCCGAC
tdbD00000505|Arabidopsis_thali  GTCGTTG.TAGTATAGT.GGTAA.GTATTCCCGCCTGTCACGCGGGTGA.CCCGGGTTCGAT.CCCCGGC
tdbD00004460|Arabidopsis_thali  GTCGTTG.TAGTATAGT.GGTAA.GGATTCCCGCCTGTCACGCGGGTGA.CCCGGGTTCGAT.CCTCGGC
tdbD00004462|Arabidopsis_thali  GTCGTTG.TAGTATAGT.GGTAA.GTATTCCCGCCTGTCACGCGGGTGA.CCCGGGTTCGAT.CCCCGGC
tdbD00000504|Arabidopsis_thali  GTCGTTG.TAGTATAGT.GGTAA.GTATTCCCGCCTGTCACCCGGGTGA.CCCGGGTTCGAT.CCCCGGC
tdbD00004461|Arabidopsis_thali  GTCGTTG.TAGTATAGT.GGTAA.GTATTCCCGCCTGTCACGCGGGTGA.CCCGGGTTCGAT.CCCCGGC
tdbD00003254|Drosophila_melano  .GTTTCCGTGGTGTAGT.GGTTA.TCACATCCGCCTAACACGCGGAAGGCCCCCGGTTCAATCCCGGGCG
tdbD00011022|Drosophila_melano  .GTTTCCGTGGTGTAGT.GGTTA.TCACATCCGCCTAACACGCGGAAGGCCCCCGGTTCAATCCCGGGCG
tdbD00011021|Drosophila_melano  .GTTTCCGTGGTGTAGC.GGTTA.TCACATCTGCCTAACACGCAGAAGGCCCCCGGTTCGATCCCGGGCG
tdbD00003259|Homo_sapiens|9606  .GTTTCCGTAGTGTAGT.GGTTA.TCACGTTCGCCTAACACGCGAAAGGTCCCCGGTTCGAAACCGGGCG
tdbD00010991|Homo_sapiens|9606  .GTTTCCGTAGTGTAGT.GGTTA.TCACGTTCGCCTAACACGCGAAAGGTCCCCGGTTCGAAACCGGGCG
tdbD00003261|Homo_sapiens|9606  .GTTTCCGTAGTGTAGT.GGTCA.TCACGTTCGCCTAACACGCGAAAGGTCCCCGGTTCGAAACCGGGCG
tdbD00010990|Homo_sapiens|9606  .GTTTCCGTAGTGTAGT.GGTCA.TCACGTTCGCCTAACACGCGAAAGGTCCCCGGTTCGAAACCGGGCG
tdbD00010987|Homo_sapiens|9606  .GTTTCCGTAGTGTAGT.GGTTA.TCACGTTTGCCTAACACGCGAAAGGTCCCCGGTTCGAAACCGGGCA
tdbD00010988|Homo_sapiens|9606  .GTTTCCGTAGTGTAGT.GGTTA.TCACGTTCGCCTAACACGCGAAAGGTCCGCGGTTCGAAACCGGGCG
tdbD00003262|Homo_sapiens|9606  .GTTTCCGTAGTGTAGT.GGTTA.TCACGTTCGCCTAACACGCGAAAGGTCCCTGGATCAAAACCAGGCG
tdbD00010989|Homo_sapiens|9606  .GTTTCCGTAGTGTAGT.GGTTA.TCACGTTCGCCTAACACGCGAAAGGTCCCTGGATCAAAACCAGGCG
tdbD00010985|Homo_sapiens|9606  .GTTTCCATAGTGTACT.GGTTA.TCACATTCACCTAACACGCGAAAGGTCCTTGGTTTGAAACCAGGCA
tdbD00003250|Arabidopsis_thali  .GGTTTCGTGGTGTAGTTGGTTA.TCACGTCAGTCTAACACACTGAAGGTCTCCGGTTCGAACCCGGGCG
```

```
                                    70
tdbD00000964|Drosophila_melano  GATGCA.
tdbD00005350|Drosophila_melano  GATGCA.
tdbD00000965|Drosophila_melano  GATGCA.
tdbD00005349|Drosophila_melano  GATGCA.
tdbD00000970|Homo_sapiens|9606  AATGCA.
tdbD00005318|Homo_sapiens|9606  AATGCA.
tdbD00005317|Homo_sapiens|9606  AGTGCA.
tdbD00005316|Homo_sapiens|9606  CATGC..
tdbD00005319|Homo_sapiens|9606  CATGCA.
tdbD00000955|Arabidopsis_thali  GGTGCA.
tdbD00005328|Arabidopsis_thali  GGTGCA.
tdbD00005327|Arabidopsis_thali  GGTGCA.
tdbD00005330|Arabidopsis_thali  GGTGCA.
tdbD00005324|Arabidopsis_thali  GGTGCA.
tdbD00005326|Arabidopsis_thali  GGTGC..
tdbD00005329|Arabidopsis_thali  GGTGC..
tdbD00000508|Drosophila_melano  GGGGAG.
tdbD00004470|Drosophila_melano  GGGGAG.
tdbD00004469|Drosophila_melano  TGGGAG.
tdbD00004468|Drosophila_melano  GGGGA..
tdbD00004458|Homo_sapiens|9606  GGGGAG.
tdbD00004454|Homo_sapiens|9606  GAGGA..
tdbD00004457|Homo_sapiens|9606  GGGGAG.
tdbD00000510|Mus_musculus|1009  GGGGAG.
tdbD00004455|Homo_sapiens|9606  GGGGA..
tdbD00004456|Homo_sapiens|9606  GGGGAG.
tdbD00004453|Homo_sapiens|9606  GGGGA..
tdbD00000505|Arabidopsis_thali  AACGGCG
tdbD00004460|Arabidopsis_thali  AACGAC.
tdbD00004462|Arabidopsis_thali  AACGGCG
tdbD00000504|Arabidopsis_thali  AACGGCG
tdbD00004461|Arabidopsis_thali  AAAGGCG
tdbD00003254|Drosophila_melano  GAAACA.
tdbD00011022|Drosophila_melano  GAAACA.
tdbD00011021|Drosophila_melano  GAAAC..
tdbD00003259|Homo_sapiens|9606  GAAACA.
tdbD00010991|Homo_sapiens|9606  GAAACA.
tdbD00003261|Homo_sapiens|9606  GAAACA.
tdbD00010990|Homo_sapiens|9606  GAAACA.
tdbD00010987|Homo_sapiens|9606  GAAACA.
tdbD00010988|Homo_sapiens|9606  GAAACA.
tdbD00003262|Homo_sapiens|9606  GAAACA.
tdbD00010989|Homo_sapiens|9606  GAAACA.
tdbD00010985|Homo_sapiens|9606  GAAAC..
tdbD00003250|Arabidopsis_thali  AAGCCA.
```
